# Optimization of non-coding regions for a non-modified mRNA COVID-19 vaccine

**DOI:** 10.1038/s41586-021-04231-6

**Published:** 2021-11-18

**Authors:** Makda S. Gebre, Susanne Rauch, Nicole Roth, Jingyou Yu, Abishek Chandrashekar, Noe B. Mercado, Xuan He, Jinyan Liu, Katherine McMahan, Amanda Martinot, David R. Martinez, Victoria Giffin, David Hope, Shivani Patel, Daniel Sellers, Owen Sanborn, Julia Barrett, Xiaowen Liu, Andrew C. Cole, Laurent Pessaint, Daniel Valentin, Zack Flinchbaugh, Jake Yalley-Ogunro, Jeanne Muench, Renita Brown, Anthony Cook, Elyse Teow, Hanne Andersen, Mark G. Lewis, Adrianus C. M. Boon, Ralph S. Baric, Stefan O. Mueller, Benjamin Petsch, Dan H. Barouch

**Affiliations:** 1 Center for Virology and Vaccine Research, Beth Israel Deaconess Medical Center, Harvard Medical School, Boston, MA USA; 2 CureVac AG, Tübingen, Germany; 3 Tufts University Cummings School of Veterinary Medicine, North Grafton, MA USA; 4 University of North Carolina at Chapel Hill, Chapel Hill, NC USA; 5 Department of Emergency Medicine, Beth Israel Deaconess Medical Center, Boston, MA USA; 6 Bioqual, Rockville, MD USA; 7 Department of Internal Medicine, Washington University School of Medicine, St. Louis, MO USA; 8 Ragon Institute of MGH, Ragon Institute of MGH, MIT and Harvard, Cambridge, MA USA

**Keywords:** SARS-CoV-2, RNA vaccines

## Abstract

The CVnCoV (CureVac) mRNA vaccine for severe acute respiratory syndrome coronavirus-2 (SARS-CoV-2) was recently evaluated in a phase 2b/3 efficacy trial in humans^1^. CV2CoV is a second-generation mRNA vaccine containing non-modified nucleosides but with optimized non-coding regions and enhanced antigen expression. Here we report the results of a head-to-head comparison of the immunogenicity and protective efficacy of CVnCoV and CV2CoV in non-human primates. We immunized 18 cynomolgus macaques with two doses of 12 μg lipid nanoparticle-formulated CVnCoV or CV2CoV or with sham (*n* = 6 per group). Compared with CVnCoV, CV2CoV induced substantially higher titres of binding and neutralizing antibodies, memory B cell responses and T cell responses as well as more potent neutralizing antibody responses against SARS-CoV-2 variants, including the Delta variant. Moreover, CV2CoV was found to be comparably immunogenic to the BNT162b2 (Pfizer) vaccine in macaques. Although CVnCoV provided partial protection against SARS-CoV-2 challenge, CV2CoV afforded more robust protection with markedly lower viral loads in the upper and lower respiratory tracts. Binding and neutralizing antibody titres were correlated with protective efficacy. These data demonstrate that optimization of non-coding regions can greatly improve the immunogenicity and protective efficacy of a non-modified mRNA SARS-CoV-2 vaccine in non-human primates.

## Abstract (web summary)

CV2CoV, a second-generation mRNA COVID-19 vaccine with non-modified nucleosides but optimized non-coding regions, is demonstrated to be effective against SARS-CoV-2 challenge when tested in non-human primates.

## Main

**Fig. 1:**
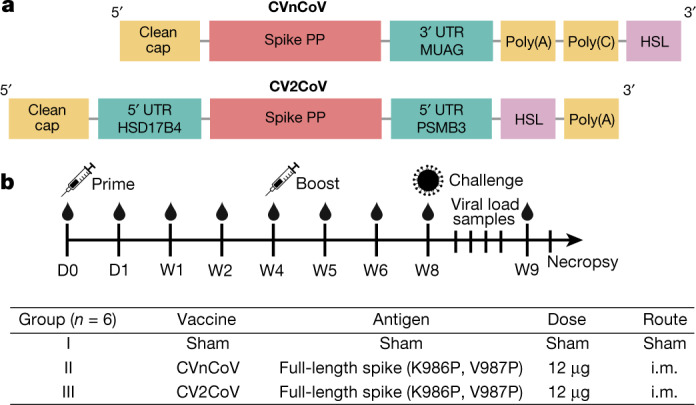
Vaccine design and study schema. **a**, Designs of the CVnCoV and CV2CoV mRNA vaccine candidates. Both vaccines are based on CureVac’s RNActive platform and encode SARS-CoV-2 spike protein with di-proline mutations. The vaccines differ in their unique non-coding regions, as shown. **b**, Non-human primate vaccine study schema. Cynomolgus macaques were immunized intramuscularly (i.m.) on day (D) 0 with CVnCoV (*n* = 6) or CV2CoV (*n* = 6) mRNA vaccine or were designated as sham (*n* = 6). The animals were boosted at week 4 and were challenged at week (W) 8. Samples were collected weekly after immunization and on days 0, 1, 2, 4, 7 and 10 after challenge for immunological and virological assays. PP, K986P and V987P mutations; HSL, histone stem–loop.

Efficacy results in humans have recently been reported for the CVnCoV (CureVac) mRNA vaccine in the phase 2b/3 HERALD trial in a population that included multiple viral variants. In this trial, the observed vaccine efficacy against symptomatic coronavirus disease 2019 (COVID-19) was approximately 48% and 53% in the overall study population and in a subgroup of participants 18–60 years of age, respectively^1^. CV2CoV is a second-generation mRNA vaccine that incorporates modifications of non-coding regions that were selected by empiric screening for improved antigen expression^2,3^. Both CVnCoV and CV2CoV are based on RNActive technology^4–7^ and consist of non-chemically modified sequence-engineered mRNA without pseudouridine^6–12^. Both vaccines encode the same full-length, pre-fusion stabilized severe acute respiratory syndrome coronavirus-2 (SARS-CoV-2) spike protein^13,14^ and are encapsulated in lipid nanoparticles (LNPs) with identical composition. CV2CoV has been engineered with different non-coding regions flanking the open reading frame, which have previously been shown to improve transgene expression^3^ and protection against SARS-CoV-2 in *ACE2*-transgenic mice^2^. Specifically, CV2CoV includes 5′ untranslated region (UTR) HSD17B4 and 3′ UTR PSMB3 elements followed by a histone stem–loop motif and a poly(A) sequence (Fig. 1 and Methods). In the present study, we make a head-to-head comparison of the immunogenicity and protective efficacy of CVnCoV and CV2CoV against SARS-CoV-2 challenge in non-human primates.

## Vaccine immunogenicity

**Extended Data Fig. 1:**
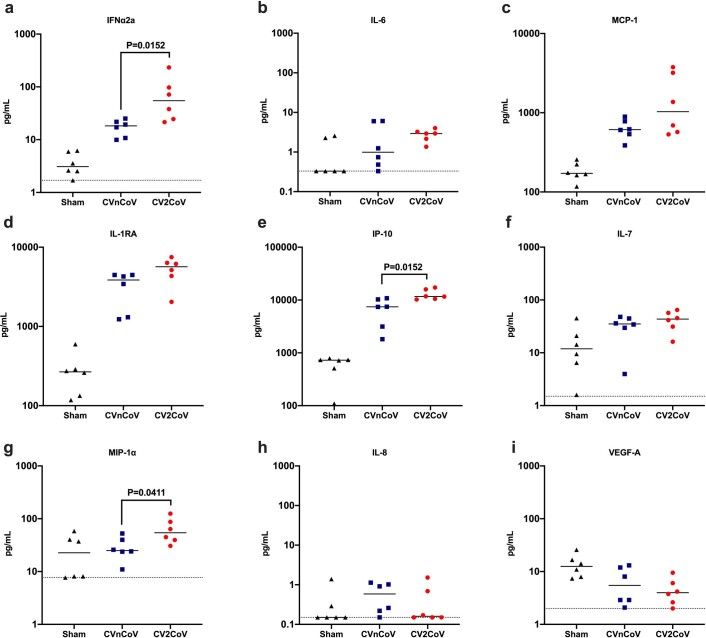
Innate cytokine induction following mRNA immunization (6/group). Sera isolated 24h post first injection were analyzed for a panel of 19 cytokines associated with viral infection using a U-PLEX Viral Combo kit from Meso Scale Discovery. Changes in cytokine levels above the detection limits were detectable for 9 cytokines. Each dot represents an individual animal, bars depict the median and the dotted line shows limit of detection. Statistical analysis was performed using two-tailed nonparametric Mann-Whitney test. Source data

We immunized 18 cynomolgus macaques intramuscularly with 12 µg CVnCoV, 12 µg CV2CoV or sham vaccine (Fig. 1b). The animals were primed at week 0 and were boosted at week 4. No clinical adverse effects were observed following vaccination. To assess innate immune responses, sera were isolated from all animals 24 h after the first vaccination to evaluate innate cytokine responses. CV2CoV induced higher levels of IFNα2a, IP-10 and MIP-1 than CVnCoV (*P* = 0.0152, *P* = 0.0152 and *P* = 0.0411, respectively; Extended Data Fig. 1).

**Fig. 2:**
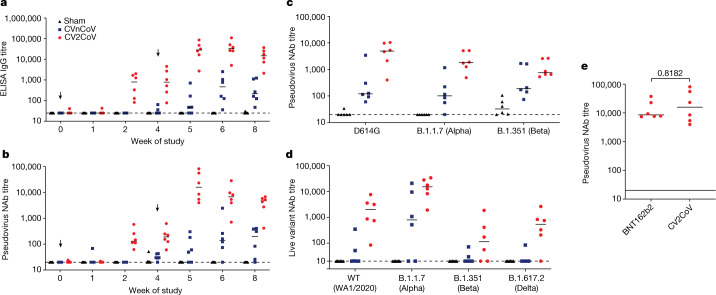
CV2CoV elicits high levels of binding and neutralizing antibody responses in macaques. Animals (*n* = 6 per group) were vaccinated twice with 12 µg of CVnCoV or CV2CoV on day 0 and on day 28 or remained untreated as negative controls (sham). **a**, **b**, Titres of RBD-binding antibodies (**a**) and pseudovirus neutralizing antibodies (NAb) against the ancestral SARS-CoV-2 strain (**b**) were evaluated at different time points after the first (weeks 0, 1, 2 and 4) and second (weeks 5, 6 and 8) vaccinations. **c**, **d**, Sera collected on day 42 (week 6) were analysed for pseudovirus (**c**) and live-virus (**d**) neutralizing antibody titres against virus with the D614G mutation and the B.1.1.7 (Alpha), B.1.351 (Beta) and B.1.617.2 (Delta) variants. **e**, Sera collected from non-human primates immunized with 12 µg of CVnCoV or 30 µg of BNT162b2 on day 35 (week 5) after boosting were analysed for pseudovirus neutralizing antibody titres against the ancestral WA/2020 (WT) strain. Each dot represents an individual animal and bars depict the median; the dashed line shows the limit of detection. Source data

Binding antibody responses were assessed by performing receptor-binding domain (RBD)-specific enzyme-linked immunosorbent assays (ELISAs) at multiple time points following immunization^15,16^. At week 2, binding antibody titres were detected only with CV2CoV and not with CVnCoV, with median values of 25 (range, 25–25) and 799 (range, 82–2,010) for CVnCoV and CV2CoV, respectively (Fig. 2a). One week after the week-4 boost, the antibody titres were increased in both groups, with medians of 48 (range, 75–710) and 28,407 (range, 2,714–86,541) for CVnCoV and CV2CoV, respectively (Fig. 2a). By week 8, the binding antibody titres had increased in the CVnCoV group but were still >50 times lower than those in the CV2CoV group (*P* = 0.0043), with median values of 214 (range, 47–1,238) and 14,827 (range, 2,133–37,079), respectively.

Neutralizing antibody responses were assessed by pseudovirus neutralization assay using the vaccine-matched SARS-CoV-2 wild-type (WT) WA1/2020 strain^15–17^. The neutralizing antibody titres followed a trend similar to that of the binding antibody titres (Fig. 2b). At week 2, neutralizing antibodies were detected only with CV2CoV and not with CVnCoV, with median values of 20 (range, 20–20) and 131 (range, 62–578) for CVnCoV and CV2CoV, respectively (Fig. 2b). One week after the week 4 boost, the neutralizing antibody titres were increased, with median values of 55 (range, 20–302) and 15,827 (range, 3,985–81,081) for CVnCoV and CV2CoV, respectively. By week 8, the neutralizing antibody titres had increased in the CVnCoV group but were still >20 times lower than those in the CV2CoV group (*P* = 0.0022), with median values of 196 (range, 20–405) and 4,752 (range, 414–6,793), respectively.

**Extended Data Fig. 2:**
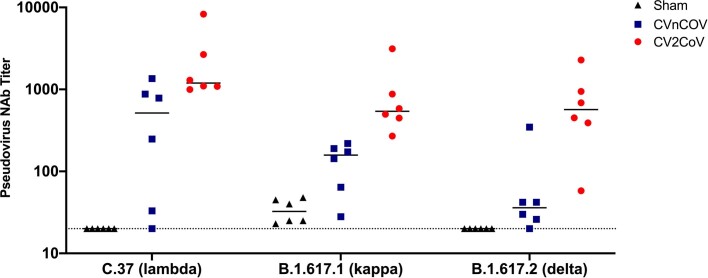
Neutralizing antibody titers against variants. Animals (6/group) were vaccinated twice with 12µg of CVnCoV or CV2CoV on d0 and d28 or remained untreated as negative controls (sham). Sera isolated on d42 (week 6) were analyzed for pseudovirus neutralizing antibody titers against C.37 (Lambda), B.1.617.1 (Kappa) and B.1.617.2 (Delta) variants. Each dot represents an individual animal, bars depict the median and the dotted line shows limit of detection. Source data

At week 6, the median pseudovirus neutralizing antibody titres against the D614G, B.1.1.7 (Alpha) and B.1.351 (Beta) variants for CVnCoV were 121, 101 and 189, respectively, while they were 4,962, 1,813 and 755 for CV2CoV (Fig. 2c). The median pseudovirus neutralizing antibody titres against the C.37 (Lambda), B.1.617.1 (Kappa) and B.1.617.2 (Delta) variants for CVnCoV were 516, 158 and 36, respectively, while they were 1,195, 541 and 568 for CV2CoV (Extended Data Fig. 2). The pseudovirus neutralizing antibody titres induced by CV2CoV were higher than those induced by CVnCoV for the WT (WA1/2020), D614G, B.1.1.7 (Alpha), B.1.351 (Beta), C.37 (Lambda), B.1.617.1 (Kappa) and B.1.617.2 (Delta) strains (*P* = 0.0043, 0.0087, 0.0043, 0.1320, 0.026, 0.0022 and 0.0043, respectively). Taken together, these data show that CV2CoV induces substantially higher pseudovirus neutralizing antibody titres against SARS-CoV-2 variants than CVnCoV.

Live-virus neutralizing antibody titres^18^ were largely consistent with those for the pseudovirus. The live-virus neutralizing antibody responses elicited by CV2CoV were higher than those elicited by CVnCoV against the WA1/2020 and B.1.617.2 (Delta) strains (*P* = 0.0466 and 0.0152, respectively), with similar trends for B.1.1.7 (Alpha) and B.1.351 (Beta) (*P* = 0.0628 and 0.1450, respectively) (Fig. 2d).

We also compared the pseudovirus neutralizing antibody titres induced in macaques by two immunizations with 12 μg of CV2CoV to those induced by two immunizations with 30 μg of the Pfizer BNT162b2 clinical vaccine obtained as leftover product from pharmacies. At peak immunity at week 5, the neutralizing antibody responses induced by CV2CoV were comparable to those induced by BNT162b2 (Fig. 2e).

**Extended Data Fig. 3:**
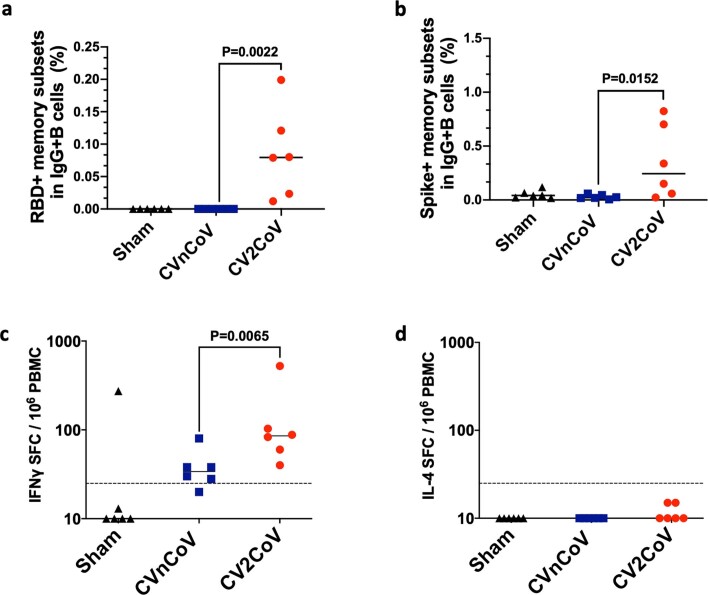
Memory B and T cell immune responses day 42 following immunization. PBMCs from negative control (sham), CVnCoV or CV2CoV vaccinated animals (6/group) isolated on d42 of the experiment were stained for (**a**) RBD and (**b**) Spike-specific activated memory B cells and analyzed by high-parameter flow cytometry. IFNγ responses to pooled spike peptides were analyzed via ELISPOT (**c**). Each dot represents an individual animal, bars depict the median and the dotted line shows limit of detection. Statistical analysis was performed using two-tailed nonparametric Mann-Whitney test. PBMC = peripheral blood mononuclear cell; SFC = spot forming cells. Source data

Most SARS-CoV-2 RBD-specific B cells reside within the memory B cell pool^19^. We used flow cytometry to assess memory B cell responses in the blood of non-human primates vaccinated with CVnCoV, CV2CoV or sham^20^. Higher numbers of RBD-specific and spike-specific memory B cells were detected in the CV2CoV-vaccinated animals as compared with those vaccinated with CVnCoV at week 6 (*P* = 0.022 and *P* = 0.0152, respectively) (Extended Data Fig. 3a, b). T cell responses were assessed by interferon γ (IFNγ) and interleukin (IL)-4 enzyme-linked immunosorbent spot (ELISPOT) assay using pooled spike peptides at week 6. IFNγ responses were detected in both groups but were higher in the CV2CoV group (*P* = 0.0065) (Extended Data Fig. 3c). IL-4 responses were not detectable, suggesting that CVnCoV and CV2CoV induce T helper type 1-biased responses (Extended Data Fig. 3d).

## Protective efficacy

**Fig. 3:**
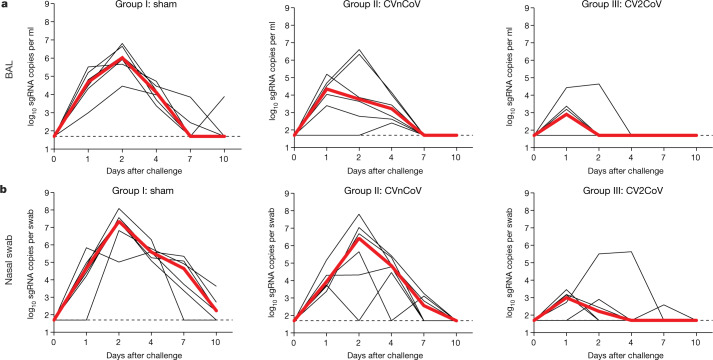
Protective efficacy of CV2CoV. Negative-control animals (sham) and animals (*n* = 6 per group) vaccinated on day 0 and day 28 with 12 µg of CVnCoV or CV2CoV were challenged with 1.0 × 10^5^ TCID_50_ of SARS-CoV-2 (USA-WA1/2020) via the intranasal and intratracheal routes. **a**, **b**, BAL (**a**) and nasal swab (**b**) samples collected on days 1, 2, 4, 7 and 10 after challenge were analysed for levels of replicating virus by RT–PCR specific for sgRNA. Thin black lines represent individual animals and thick red lines depict the median; the dashed line shows the limit of detection. Source data

**Fig. 4:**
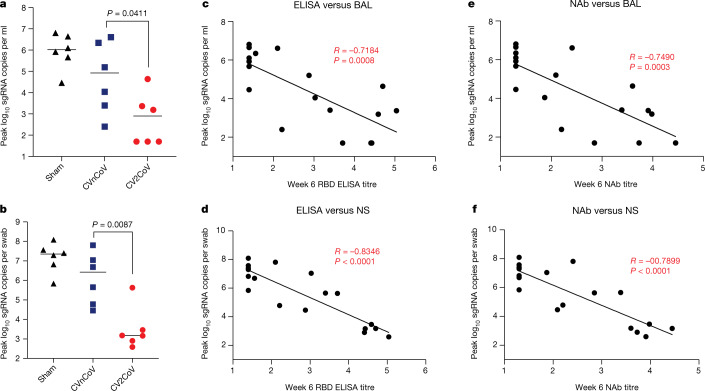
Titres of binding and neutralizing antibodies elicited following CVnCoV and CV2CoV vaccination (*n* = 6 per group) correlate with protection against SARS-CoV-2. **a**, **b**, Summary of peak viral loads following SARS-CoV-2 challenge in BAL and nasal swab (NS) samples. Animals were challenged with 1.0 × 10^5^ TCID_50_ of SARS-CoV-2 derived from strain USA-WA1/2020 (NR-52281, BEI Resources). **c–f**, Antibody correlates of protection for binding antibodies (**c**, **d**) and neutralizing antibodies (**e**, **f**). Statistical analysis was performed using the two-tailed non-parametric Mann–Whitney test, and correlation was analysed by two-sided Spearman rank-correlation test. The bars indicate median values. Source data

All animals were challenged at week 8 with 1.0 × 10^5^ median tissue culture infectious doses (TCID_50_) of the SARS-CoV-2 WA1/2020 strain via the intranasal and intratracheal routes. Viral loads were assessed in bronchoalveolar lavage (BAL) and nasal swab samples collected on days 1, 2, 4, 7 and 10 following challenge by quantitative PCR with reverse transcription (RT–PCR) specific for subgenomic RNA (sgRNA)^21^. The sgRNA levels in the BAL and nasal swab samples in the sham group peaked on day 2 and largely resolved by day 10. The sham controls had peak medians of 6.02 (range, 4.62–6.81) log_10_-transformed sgRNA copies per ml in the BAL and 7.35 (range, 5.84–8.09) log_10_-transformed sgRNA copies per swab in the nasal swab samples on day 2 (Fig. 3). The CVnCoV-immunized animals showed peak medians of 4.92 (range, 2.40–6.61) log_10_-transformed sgRNA copies per ml in the BAL and 6.42 (range, 4.46–7.81) log_10_-transformed sgRNA copies per swab in the nasal swab samples (Fig. 3). The CV2CoV-immunized animals exhibited peak medians of 2.90 (range, 1.70–4.64) log_10_-transformed sgRNA copies per ml in the BAL and 3.17 (range, 2.59–5.63) log_10_-transformed sgRNA copies per swab in the nasal swab samples (Fig. 3), with resolution of sgRNA levels in the BAL samples by day 2 in most animals and by day 4 in all animals. Overall, CV2CoV resulted in significantly lower peak viral loads than CVnCoV in both the BAL (*P* = 0.0411) and nasal swab (*P* = 0.0087) samples (Fig. 4a, b).

**Extended Data Fig. 4:**
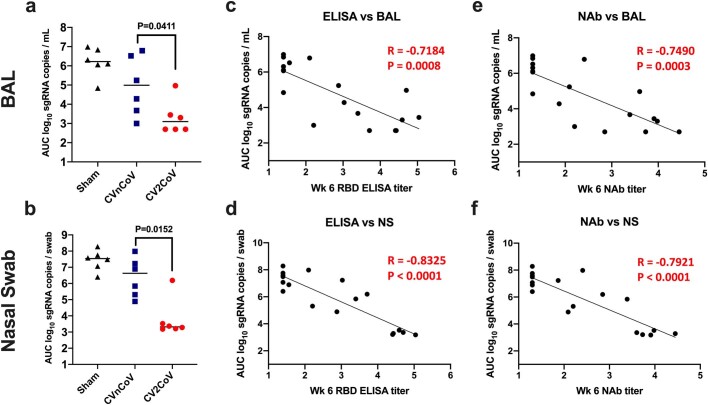
Binding and neutralizing antibody titers correlate with protection against SARS-CoV-2. Summary of area under curve (AUC) viral load values following SARS-CoV-2 challenge in BAL and nasal swab samples (6/group) (**a**, **b**); antibody correlates of protection for binding antibodies (**c**, **d**) and neutralizing antibodies (**e**, **f**). Statistical analysis was performed using two-tailed nonparametric Mann-Whitney test. Correlations was analyzed by two-sided Spearman rank-correlation test. NAbs = neutralizing antibodies, BAL = bronchoalveolar lavage NS = nasal swab. Source data

**Extended Data Fig. 5:**
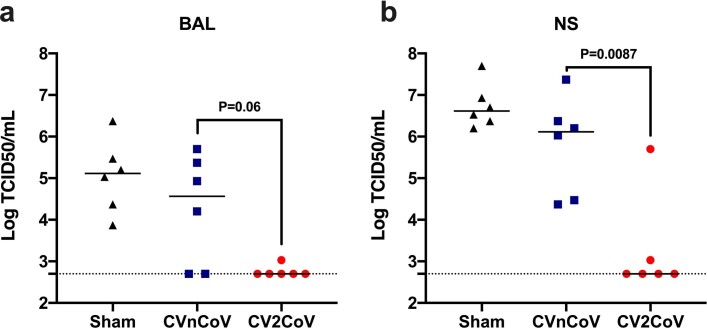
Infectious virus titers after SARS-CoV-2 challenge (6/group). Infectious virus titers of BAL and nasal swab samples collected 2 days post challenge were analyzed by TCID_50_ assays. Each dot represents an individual animal, bars depict the median and the dotted line shows limit of detection. Statistical analysis was performed using two-tailed nonparametric Mann-Whitney test. Source data

We next evaluated the immune correlates of protection. The log_10_-transformed ELISA and neutralizing antibody titres at week 6 were inversely correlated with the peak log_10_-transformed sgRNA copies per ml in the BAL samples (*P* = 0.0008, *R* = −0.7148 and *P* = 0.0015, *R* = −0.6912, respectively, by two-sided Spearman rank-correlation test) (Fig. 4c, e) and with the peak sgRNA copies per nasal swab in the nasal swab samples (*P* < 0.0001, *R* = −0.8346 and *P* < 0.0001, *R* = −0.8766, respectively, by two-sided Spearman rank-correlation test) (Fig. 4d, f). Consistent with prior observations from our laboratory and others^15,16,22^, these findings suggest that binding and neutralizing antibody titres are important correlates of protection for these SARS-CoV-2 vaccines in non-human primates. Similar correlates of protection were observed with viral loads assessed as area under the curve (Extended Data Fig. 4). Moreover, we assessed infectious virus titres by TCID_50_ assay on day 2 after challenge, which showed no detectable virus in five of six animals in the CV2CoV group (Extended Data Fig. 5).

**Extended Data Fig. 6:**
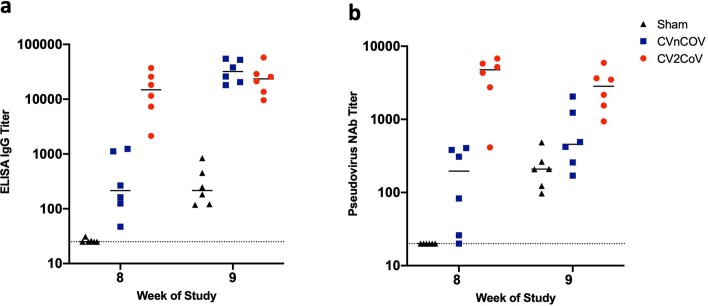
Post-challenge binding and neutralizing antibody responses (6/group). Negative control (sham) or animals vaccinated on d0 and d28 of the experiment with 12 µg of CVnCoV or CV2CoV as indicated were subjected to challenge infection using 1.0×10^5^ TCID_50_ SARS-CoV-2 via intranasal (IN) and intratracheal (IT) routes. (**a**) Titers of RBD binding antibodies and (**b**) pseudovirus neutralizing antibodies against ancestral SARS-CoV-2 strain were evaluated before (week 8) and a week after challenge infection (week 9). Each dot represents an individual animal, bars depict the median and the dotted line shows limit of detection. Statistical analysis was performed using two-tailed nonparametric Mann-Whitney test. NAbs = neutralizing antibodies. Source data

**Extended Data Fig. 7:**
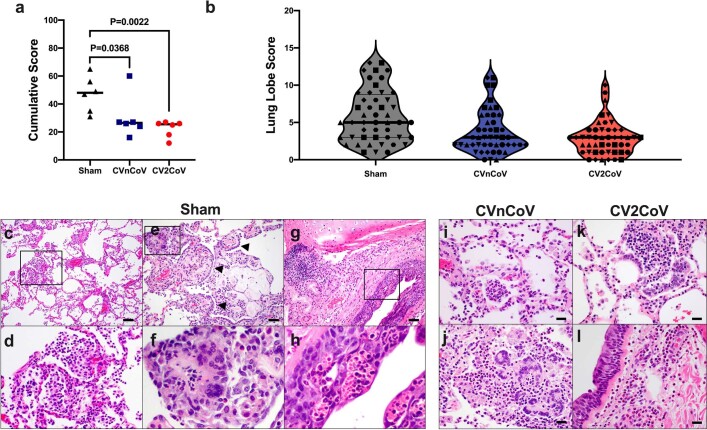
CVnCoV and CV2CoV protect the lungs from pathological changes upon viral challenge (6/group). Eight lung lobes (4 sections from right and left, caudal to cranial) were assessed and scored (1-4) for each of the following lesions: 1) Interstitial inflammation and septal thickening 2) Eosinophilic interstitial infiltrate 3) Neutrophilic interstitial infiltrate 4) Hyaline membranes 5) Interstitial fibrosis 6) Alveolar infiltrate, macrophage 7) Alveolar/Bronchoalveolar infiltrate, neutrophils 8) Syncytial cells 9) Type II pneumocyte hyperplasia 10) Broncholar infiltrate, macrophage 11) Broncholar infiltrate, neutrophils 12) BALT hyperplasia 13) Bronchiolar/peribronchiolar inflammation 14) Perivascular, mononuclear infiltrates 15) Vessels, endothelialitis. Each feature assessed was assigned a score of 0 = no significant findings; 1 = minimal; 2 = mild; 3 = moderate; 4 = marked/severe. (**a**) Cumulative scores per animal (**b**) Cumulative scores per lung lobe. Individual animals are represented by symbols. Representative histopathology from sham vaccinated (**c**-**h**), CnVCoV vaccinated (i, j), and Cv2CoV vaccinated (**k**, **l**) animals showing (c, d, inset) alveolar macrophage infiltrate, (e, f, inset) syncytial cells (arrowheads) and type II pneumocyte hyperplasia, inset (g, h, inset) bronchiolar epithelial necrosis with neutrophilic infiltrates (**i**) alveolar neutrophilic infiltrate and alveolar septal thickening (**j**) focal consolidation with inflammation composed of macrophages, neutrophils, and syncytial cells (**k**) focal pneumocyte hyperplasia, syncytial cells and inflammatory infiltrates (**l**) peribronchiolar inflammation. Statistical analysis was performed using two-tailed nonparametric Mann-Whitney test. Scale bars: 100 microns (**c**), 50 microns (**e**, **g**) 20 microns (i-l). BALT bronchus associated lymphoid tissue. Source data

Following challenge, we observed anamnestic binding and neutralizing antibody responses in all CVnCoV-vaccinated animals and in a subset of the CV2CoV-vaccinated animals^16^ (Extended Data Fig. 6). On day 10 after challenge, the animals were necropsied, and their lung tissues were evaluated by histopathology. Viral replication was largely resolved by day 10 in the animals vaccinated with CVnCoV and CV2CoV, and those with sham treatment had higher cumulative lung pathology scores^19^ (CVnCoV animals compared with sham controls, *P* = 0.0368; CV2CoV animals compared with sham controls, *P* = 0.0022) (Extended Data Fig. 7a). Animals in the sham group also had more lung lobes affected (Extended Data Fig. 7b) and more extensive lung lesions, with a greater proportion of lung lobes showing evidence of interstitial inflammation, alveolar inflammatory infiltrates and type II pneumocyte hyperplasia (Extended Data Fig. 7c–h). No significant eosinophilia was observed. The pathological lesions in vaccinated animals were similar to those observed for animals in the sham group (Extended Data Fig. 7i–l) but were overall fewer in number and more focal in distribution.

## Discussion

CV2CoV elicited substantially greater humoral and cellular immune responses and provided significantly improved protective efficacy against SARS-CoV-2 challenge as compared with CVnCoV in macaques. These data suggest that optimization of non-coding elements of the mRNA backbone can substantially improve the immunogenicity and protective efficacy of mRNA vaccines. Both CVnCoV and CV2CoV contain only non-modified nucleosides with no pseudouridine or derivates, and CV2CoV has previously been shown to lead to higher antigen expression than CVnCoV in cell culture^3^. The neutralizing antibody titres induced by CV2CoV were comparable in macaques to those induced by the clinical BNT162b2 vaccine, which incorporates pseudouridine. These results suggest that strategies other than nucleoside modification can also markedly improve mRNA potency.

Previous studies with rodents and non-human primates have demonstrated protection by CVnCoV^2,23,24^. However, protection in macaques was primarily observed in the lower respiratory tract^23,24^. In the present study, CVnCoV provided only modest viral load reductions in BAL and nasal swab samples compared with sham controls. In contrast to CVnCoV, CV2CoV induced >10-fold-higher neutralizing antibody responses against multiple viral variants and provided >3 log reductions in sgRNA copies per ml in BAL and >4 log reductions in sgRNA copies per swab in nasal swab samples compared with sham controls.

Previous mRNA vaccine clinical trials have demonstrated onset of protective efficacy after the first dose with improved protection after the boost immunization^25,26^. In the present study, the prime immunization with CV2CoV induced binding and neutralizing antibodies in all macaques by week 2, and these responses had increased substantially by 1 week after the boost immunization. The neutralizing antibody titres induced by CV2CoV in this study also appear to be similar to those reported for other mRNA vaccines in macaques^27,28^. Moreover, the neutralizing antibody titres induced by BNT162b2 in our study (Fig. 2e) were comparable to those reported for BNT162b2 in a prior study^28^.

As previously reported for other vaccines^29–33^, the neutralizing antibody titres against certain SARS-CoV-2 variants, such as the B.1.351 (Beta) and B.1.617.2 (Delta) variants, were lower than those against the parental strain WA1/2020. Although our challenge virus in this study was SARS-CoV-2 WA1/2020, the neutralizing antibody titres elicited by CV2CoV to viral variants exceeded the values we previously reported as threshold titres for protection (50–100)^17,19,22^. However, future studies will be required to directly assess the protective efficacy of CV2CoV against SARS-CoV-2 variants of concern in non-human primates.

CV2CoV induced both antigen-specific memory B cell responses and T cell responses. Although the correlates of protection in this study were binding and neutralizing antibody titres^34,35^, it is likely that CD8^+^ T cells contribute to viral clearance in tissues^36,37^. We previously reported that depletion of CD8^+^ T cells partially abrogated protective efficacy against SARS-CoV-2 re-challenge in convalescent macaques^22^. Memory B cells might contribute to the durability of antibody responses^38,39^; B cell germinal centre responses and the durability of protective efficacy following CV2CoV vaccination remain to be determined. Moreover, although this study was not specifically designed as a safety study, it is worth noting that we did not observe any adverse effects following CVnCoV or CV2CoV vaccination, nor did we observe unexpected or enhanced pathology in the vaccinated animals at necropsy^40^.

In summary, our data show that optimization of non-coding regions in a SARS-CoV-2 mRNA vaccine can substantially improve its immunogenicity against multiple viral variants and can enhance its protective efficacy against SARS-CoV-2 challenge in macaques. The improved characteristics of CV2CoV over those of CVnCoV might translate into increased efficacy in humans; accordingly, clinical trials of CV2CoV are planned.

## Methods

### mRNA vaccines

The two mRNA vaccines, CVnCoV and CV2CoV, are based on CureVac’s RNActive platform (claimed and described in, for example, patents WO2002098443 and WO2012019780) and include no chemically modified nucleosides. They are composed of a 5′ cap1 structure, a G+C-enriched open reading frame, a 3′ UTR and a vector-encoded poly(A) stretch. CVnCoV contains a cleanCap (Trilink) and parts of the 3′ UTR of the *Homo sapiens* alpha-haemoglobin gene as the 3′ UTR, followed by a poly(A)_64_ stretch, a poly(C)_30_ stretch and a histone stem–loop^22,23^. CV2CoV has previously been described and contains a cleanCap followed by the 5′ UTR from the human hydroxysteroid 17-beta dehydrogenase 4 gene (*HSD17B4*) and a 3′ UTR from the human proteasome 20S subunit beta 3 gene (*PSMB3*) followed by a histone stem–loop and a poly(A)_100_ stretch^3^. The constructs were encapsulated in LNP by Acuitas Therapeutics (CV2CoV) or Polymun Scientific Immunbiologische Forschung (CVnCoV). LNPs are composed of ionizable amino lipid, phospholipid and cholesterol and PEGylated lipid; the compositions for CVnCoV and CV2CoV are identical. Both mRNAs encode SARS-CoV-2 full-length spike protein containing stabilizing K986P and V987P mutations (NCBI reference sequence NC045512.2).

### Animals and study design

Eighteen cynomolgus macaques of both sexes between the ages of 3 and 20 years were randomly assigned to three groups. The animals received either CVnCoV (*n* = 6) or CV2CoV (*n* = 6) mRNA vaccine or were designated as sham controls (*n* = 6). The mRNA vaccines were administered intramuscularly at a 12-µg dose in the left quadriceps on day 0. Boost immunizations were similarly administered at week 4. At week 8, all animals were challenged with 1.0 × 10^5^ TCID_50_ of SARS-CoV-2 derived from USA-WA1/2020 (NR-52281, BEI Resources)^17^. The challenge virus was administered as 1 ml by the intranasal route (0.5 ml in each naris) and 1 ml by the intratracheal route. All animals were killed 10 d after challenge. Immunological and virological assays were performed with blinding. All animals were housed at Bioqual. All animal studies were conducted in compliance with all relevant local, state and federal regulations and were approved by the Bioqual Institutional Animal Care and Use Committee.

### Cytokine analyses

The serum levels of 19 analytes that have been associated with immune response to viral infection were tested using the U-PLEX Viral Combo 1 (NHP) kit (K15069L-1) obtained from Meso Scale Discovery. The 19 analytes and their detection limits (LLODs) included G-CSF (1.5 pg ml^−1^), GM-CSF (0.12 pg ml^−1^), IFNα2a (1.7 pg ml^−1^), IFNγ (1.7 pg ml^−1^), IL-1RA (1.7 pg ml^−1^), IL-1β (0.15 pg ml^−1^), IL-4 (0.06 pg ml^−1^), IL-5 (0.24 pg ml^−1^), IL-6 (0.33 pg ml^−1^), IL-7 (1.5 pg ml^−1^), IL-8 (0.15 pg ml^−1^), IL-9 (0.14 pg ml^−1^), IL-10 (0.14 pg ml^−1^), IL-12p70 (0.54 pg ml^−1^), IP-10 (0.49 pg ml^−1^), MCP-1 (0.74 pg ml^−1^), MIP-1α (7.7 pg ml^−1^), TNF (0.54 pg ml^−1^) and VEGF-A (2.0 pg ml^−1^). All serum samples were assayed in duplicate. The assay was performed by the Metabolism and Mitochondrial Research Core (Beth Israel Deaconess Medical Center, Boston, MA) following the manufacture’s instructions. The assay plates were read by a MESO QuickPlex SQ 120 instrument, and the data were analysed using Discovery Workbench 4.0 software.

### ELISA

RBD-specific binding antibodies were assessed by ELISA as described previously^16,17^. In brief, 96-well plates were coated with 1 μg ml^−1^ SARS-CoV-2 RBD protein (40592-VNAH, Sino Biological) in 1× DPBS and were incubated at 4 °C overnight. After incubation, the plates were washed once with wash buffer (0.05% Tween-20 in 1× DPBS) and were blocked with 350 μl casein block per well for 2–3 h at room temperature. After incubation, the block solution was discarded, and the plates were blotted dry. Serial dilutions of heat-inactivated serum diluted in casein block were added to the wells, and the plates were incubated for 1 h at room temperature. Next, the plates were washed three times and were then incubated for 1 h with a 1:1,000 dilution of anti-macaque IgG HRP (NIH NHP Reagent Program) at room temperature in the dark. The plates were then washed three more times, and 100 μl of SeraCare KPL TMB SureBlue Start solution was added to each well; plate development was halted by the addition of 100 μl of SeraCare KPL TMB Stop solution per well. The absorbance at 450 nm was recorded using a VersaMax or Omega microplate reader. The ELISA endpoint titres were defined as the highest reciprocal serum dilution that yielded an absorbance >0.2, and the log_10_ endpoint titres are reported. The immunological assays were performed with blinding.

### Pseudovirus neutralization assay

SARS-CoV-2 pseudoviruses encoding a luciferase reporter gene were generated as described previously^15^. In brief, the packaging plasmid psPAX2 (AIDS Resource and Reagent Program), luciferase reporter plasmid pLenti-CMV Puro-Luc (Addgene) and spike protein-encoding pcDNA3.1-SARS CoV-2 SΔCT plasmid of variants were co-transfected into HEK293T cells by Lipofectamine 2000 (ThermoFisher Scientific). Pseudoviruses of SARS-CoV-2 variants were generated by using the WA1/2020 strain (Wuhan/WIV04/2019; GISAID accession ID, EPI_ISL_402124), the strain with a D614G mutation, the B.1.1.7 variant (GISAID accession ID, EPI_ISL_601443), the B.1.351 variant (GISAID accession ID, EPI_ISL_712096), the C37 variant (GenBank ID, QRX62290), the B.1.671.1 variant (GISAID accession ID, EPI_ISL_1384866) and the B.1.617.2 variant (GISAID accession ID, EPI_ISL_2020950). Supernatants containing the pseudotype viruses, which were purified by centrifugation and filtration with a 0.45-µm filter, were collected 48 h after transfection. To determine the neutralization activity of the plasma or serum samples from the animals studied, HEK293T-hACE2 cells were seeded in 96-well tissue culture plates at a density of 1.75 × 10^4^ cells per well overnight. Threefold serial dilutions of heat-inactivated serum or plasma samples were prepared and mixed with 50 µl of pseudovirus. The mixture was incubated at 37 °C for 1 h before being added to the HEK293T-hACE2 cells. The cells were lysed 48 h after infection in Steady-Glo Luciferase Assay buffer (Promega) according to the manufacturer’s instructions. The SARS-CoV-2 neutralization titres were defined as the sample dilution at which a 50% reduction in relative light units (RLU) was observed relative to the average of the virus control wells.

### Live-virus neutralization assay

Full-length SARS-CoV-2 WA1/2020, B.1.1.7, B.1.351 and B.1.617.2 viruses were designed to encode nanoluciferase (nLuc) and were recovered via reverse genetics^18^. One day before the assay, Vero E6 USAMRID cells were plated at 20,000 cells per well in clear-bottomed, black-walled plates. The cells were inspected to ensure confluency on the day of the assay. The serum samples were tested at a starting dilution of 1:20 and were serially diluted threefold for up to nine dilution spots. The serially diluted serum samples were mixed with diluted virus in an equal volume. The antibody–virus and virus-only mixtures were then incubated at 37 °C with 5% CO_2_ for 1 h. After incubation, the serially diluted sera and virus-only controls were added in duplicate to the cells at 75 plaque-forming units at 37 °C with 5% CO_2_. The cells were lysed 24 h later, and the luciferase activity was measured via Nano-Glo Luciferase Assay System (Promega) according to the manufacturer’s specifications. The luminescence was measured by a Spectramax M3 plate reader (Molecular Devices). Virus neutralization titres were defined as the sample dilution at which a 50% reduction in RLU was observed relative to the average of the virus control wells.

### B cell immunophenotyping

Fresh peripheral blood mononuclear cells were stained with Aqua live/dead dye (Invitrogen) for 20 min, washed with 2% FBS in DPBS and suspended in 2% FBS in DPBS with Fc Block (BD) for 10 min, followed by staining with monoclonal antibodies against CD45 (clone D058-1283, BUV805), CD3 (clone SP34.2, APC-Cy7), CD7 (clone M-T701, Alexa700), CD123 (clone 6H6, Alexa700), CD11c (clone 3.9, Alexa700), CD20 (clone 2H7, PE-Cy5), IgA (goat polyclonal antibodies, APC), IgG (clone G18-145, BUV737), IgM (clone G20-127, BUV396), IgD (goat polyclonal antibodies, PE), CD80 (clone L307.4, BV786), CD95 (clone DX2, BV711), CD27 (clone M-T271, BUV563), CD21 (clone B-ly4, BV605), CD14 (clone M5E2, BV570) and CD138 (clone DL-101, PE-CF594). The cells were also stained for SARS-CoV-2 antigens including biotinylated SARS-CoV-2 RBD protein (Sino Biological) and full-length SARS-CoV-2 spike protein (Sino Biological) labelled with FITC and DyLight 405 (DyLight 405 Conjugation Kit and FITC Conjugation Kit, Abcam) at 4 °C for 30 min. After staining, the cells were washed twice with 2% FBS in DPBS, incubated with BV650 streptavidin (BD Pharmingen) for 10 min and then washed twice with 2% FBS in DPBS. After staining, the cells were washed and fixed with 2% paraformaldehyde. All data were acquired on a BD FACSymphony flow cytometer. Subsequent analyses were performed using FlowJo software (Treestar, v.9.9.6). The immunological assays were performed with blinding.

### IFNγ ELISPOT assay

ELISPOT plates were coated with mouse anti-human IFNγ monoclonal antibody (BD Pharmingen) at a concentration of 5 μg per well overnight at 4 °C. The plates were washed with DPBS containing 0.25% Tween-20 and were blocked with R10 medium (RPMI with 11% FBS and 1.1% penicillin–streptomycin) for 1 h at 37 °C. The S1 and S2 peptide pools (custom made, JPT Peptide Technologies) used in the assay contained peptides of 15 amino acids in length, overlapping by 11 amino acids, that spanned the protein sequence and reflect the N-terminal and C-terminal halves of the protein, respectively. The S1 and S2 peptide pools were prepared at a concentration of 2 μg per well, and 200,000 cells per well were added. The peptides and cells were incubated for 18–24 h at 37 °C. All steps following this incubation were performed at room temperature. The plates were washed with ELISPOT wash buffer and were incubated for 2 h with 1 μg ml^−1^ rabbit polyclonal anti-human IFNγ biotin obtained from U-Cytech. The plates were washed a second time and were then incubated for 2 h with 1 μg ml^−1^ streptavidin–alkaline phosphatase antibody obtained from Southern Biotech. The final wash was followed by the addition of nitro-blue tetrazolium chloride/5-bromo-4-chloro 3′ indolyl phosphate *p*-toludine salt (NBT/BCIP chromagen) substrate solution (ThermoFisher Scientific) for 7 min. The chromogen was discarded, and the plates were washed with water and were dried in a dim location for 24 h. The plates were then scanned and counted using an ELISPOT analyser (Immunospot).

### IL-4 ELISPOT assay

ELISPOT plates precoated with monoclonal antibody against IL-4 (Mabtech) were washed and blocked. The assay was then performed as described above except that the development time with NBT/BCIP chromagen substrate solution was 12 min.

### Subgenomic RT–PCR assay

SARS-CoV-2 *E* gene sgRNA was assessed by RT–PCR using primers and probes as previously described^15,17^. A standard was generated by first synthesizing a gene fragment of the subgenomic *E* gene. The gene fragment was subsequently cloned into the pcDNA3.1+ expression plasmid using restriction site cloning (Integrated DNA Technologies). The insert was transcribed in vitro to RNA using the AmpliCap-Max T7 High Yield Message Maker kit (CellScript). Log dilutions of the standard were prepared for RT–PCR assays, ranging from 1 ×10^10^ copies to 1 ×10^−1^ copies. The viral loads were quantified from BAL fluid and nasal swab samples. RNA extraction was performed on a QIAcube HT using the IndiSpin QIAcube HT Pathogen kit according to the manufacturer’s specifications (Qiagen). The standard dilutions and extracted RNA samples were reverse-transcribed using SuperScript VILO Master Mix (Invitrogen) following the cycling conditions described by the manufacturer. A Taqman custom gene expression assay (ThermoFisher Scientific) was designed using the sequences targeting the *E* gene sgRNA. The sequences for the custom assay were as follows: forward primer, sgLeadCoV2.Fwd: 5′-CGATCTCTTGTAGATCTGTTCTC-3′; E_Sarbeco_R: 5′-ATATTGCAGCAGTACGCACACA-3′; E_Sarbeco_P1 (probe): 5′-VIC-ACACTAGCCATCCTTACTGCGCTTCG-MGBNFQ-3′. Reactions were carried out in duplicate for samples and standards on QuantStudio 6 and 7 Flex Real-Time PCR systems (Applied Biosystems) with the following thermal cycling conditions: initial denaturation at 95 °C for 20 s followed by 45 cycles of 95 °C for 1 s and 60 °C for 20 s. Standard curves were used to calculate the sgRNA copies per millilitre or per swab. The quantitative assay sensitivity was determined as 50 copies per millilitre or per swab.

### TCID_50_ assay

Vero TMPRSS2 cells (obtained from A. Creanga, NIH) were plated at 25,000 cells per well in DMEM with 10% FBS and gentamicin, and the cultures were incubated at 37 °C, 5.0% CO_2_. Medium was aspirated and replaced with 180 μl of DMEM with 2% FBS and gentamicin. Serial dilution of samples as well as positive (virus stock of known infectious titre) and negative (medium only) controls were included in each assay. The plates were incubated at 37 °C, 5.0% CO_2_, for 4 d, and the cell monolayers were visually inspected for cytopathic effects. TCID_50_ was calculated using the Read–Muench formula.

### Histopathology

At the time of fixation, lungs were suffused with 10% formalin to expand the alveoli. All tissues were fixed in 10% formalin and block-sectioned at 5 µm. The slides were baked for 30–60 min at 65 °C, deparaffinized in xylene, rehydrated through a series of graded ethanol to distilled water and then stained with haematoxylin and eosin. Blinded histopathological evaluation was performed by a board-certified veterinary pathologist (A.J.M.).

### Statistical analyses

Statistical analyses were performed using GraphPad Prism (version 9.0) software (GraphPad Software), and comparisons between groups were performed using a two-tailed non-parametric Mann–Whitney *U* test. *P* values of less than 0.05 were considered as significant. Correlations were assessed by applying two-sided Spearman rank-correlation tests.

### Reporting summary

Further information on research design is available in the Nature Research Reporting Summary linked to this paper.

## Online content

Any methods, additional references, Nature Research reporting summaries, source data, extended data, supplementary information, acknowledgements, peer review information; details of author contributions and competing interests; and statements of data and code availability are available at 10.1038/s41586-021-04231-6.

## Supplementary information

Reporting Summary

## Data Availability

All data are available in the manuscript and its Supplementary Information. Source data are provided with this paper.

## Source data

Source Data Fig. 2
Source Data Fig. 3
Source Data Fig. 4
Source Data Extended Data Fig. 1
Source Data Extended Data Fig. 2
Source Data Extended Data Fig. 3
Source Data Extended Data Fig. 4
Source Data Extended Data Fig. 5
Source Data Extended Data Fig. 6
Source Data Extended Data Fig. 7
