# Supplementary material for: Optimization of non-coding regions for a non-modified mRNA COVID-19 vaccine
Source: Nature. 2021 Nov 18;601(7893):410–4. doi: 10.1038/s41586-021-04231-6 (PMC8770133; doi:10.1038/s41586-021-04231-6)
Supplement: Supplementary file 1 — Reporting Summary [file 41586_2021_4231_MOESM1_ESM.pdf]

nature portfolio

Corresponding author(s): Dan Barouch 2021-08-13132

Last updated by author(s): Nov 7, 2021

Reporting Summary

Nature Portfolio wishes to improve the reproducibility of the work that we publish. This form provides structure for consistency and transparency in reporting. For further information on Nature Portfolio policies, see our Editorial Policies and the Editorial Policy Checklist.

Statistics

For all statistical analyses, confirm that the following items are present in the figure legend, table legend, main text, or Methods section.

n/a	Confirmed	
☐	☒	The exact sample size (n) for each experimental group/condition, given as a discrete number and unit of measurement
☐	☒	A statement on whether measurements were taken from distinct samples or whether the same sample was measured repeatedly
☐	☒	The statistical test(s) used AND whether they are one- or two-sided<br>*Only common tests should be described solely by name; describe more complex techniques in the Methods section.*
☐	☒	A description of all covariates tested
☐	☒	A description of any assumptions or corrections, such as tests of normality and adjustment for multiple comparisons
☐	☒	A full description of the statistical parameters including central tendency (e.g. means) or other basic estimates (e.g. regression coefficient) AND variation (e.g. standard deviation) or associated estimates of uncertainty (e.g. confidence intervals)
☐	☒	For null hypothesis testing, the test statistic (e.g. F, t, r) with confidence intervals, effect sizes, degrees of freedom and P value noted<br>*Give P values as exact values whenever suitable.*
☒	☐	For Bayesian analysis, information on the choice of priors and Markov chain Monte Carlo settings
☒	☐	For hierarchical and complex designs, identification of the appropriate level for tests and full reporting of outcomes
☐	☒	Estimates of effect sizes (e.g. Cohen's d, Pearson's r), indicating how they were calculated

Our web collection on statistics for biologists contains articles on many of the points above.

Software and code

Policy information about availability of computer code

Data collection	Flow Jo Version 10.6.2 was used
Data analysis	Virologic and immunologic data analysis was performed using GraphPad Prism 9.0.0 (GraphPad Software). Specifically, cytokine data analysis was done using DISCOVERY WORKBENCH® 4.0 software.

For manuscripts utilizing custom algorithms or software that are central to the research but not yet described in published literature, software must be made available to editors and reviewers. We strongly encourage code deposition in a community repository (e.g. GitHub). See the Nature Portfolio guidelines for submitting code & software for further information.

Data

Policy information about availability of data

All manuscripts must include a data availability statement. This statement should provide the following information, where applicable:

- Accession codes, unique identifiers, or web links for publicly available datasets
- A description of any restrictions on data availability
- For clinical datasets or third party data, please ensure that the statement adheres to our policy

All data are available in the manuscript and the supplementary material.

Field-specific reporting

Please select the one below that is the best fit for your research. If you are not sure, read the appropriate sections before making your selection.

☒ Life sciences ☐ Behavioural & social sciences ☐ Ecological, evolutionary & environmental sciences

For a reference copy of the document with all sections, see nature.com/documents/nr-reporting-summary-flat.pdf

Life sciences study design

All studies must disclose on these points even when the disclosure is negative.

Sample size	Sample size includes N=18 animals (N=6 animals for each vaccine or sham control groups). Based on our previous experience with SARS-CoV-2 in cynomolgus as well as rhesus macaques, this sample size provides sufficient power to determine differences in protective efficacy of both vaccinated groups compared with the sham controls.
Data exclusions	No data were excluded.
Replication	Virologic and immunologic measures were performed in duplicate. Technical replicates were minimally different. Attempts in replication were succesful
Randomization	Animals were balanced for age and gender and otherwise randomly allocated to groups. All other sample randomization through out the study was random.
Blinding	All immunologic and virologic assays were performed blinded.

Reporting for specific materials, systems and methods

We require information from authors about some types of materials, experimental systems and methods used in many studies. Here, indicate whether each material, system or method listed is relevant to your study. If you are not sure if a list item applies to your research, read the appropriate section before selecting a response.

Materials & experimental systems

n/a	Involved in the study
☐	☒ Antibodies
☐	☒ Eukaryotic cell lines
☒	☐ Palaeontology and archaeology
☐	☒ Animals and other organisms
☒	☐ Human research participants
☒	☐ Clinical data
☒	☐ Dual use research of concern

Methods

n/a	Involved in the study
☒	☐ ChIP-seq
☒	☐ Flow cytometry
☒	☐ MRI-based neuroimaging

Antibodies

Antibodies used	For ELISA assay: anti-macaque IgG HRP (NIH NHP Reagent Program); for ELISPOT assay: mouse anti-human IFN-γ monoclonal antibody (BD Pharmingen), IL-4 capture monoclonal antibody (Mabtech), rabbit polyclonal anti-human IFN-γ Biotin (U-Cytech) and Streptavidin-alkaline phosphatase antibody (IFN-γ: Southern Biotechnology/IL-4 (Mabtech). For B cell ICS assay: monoclonal antibodies against CD45 (clone D058-1283, BUV805), CD3 (clone SP34.2 , APC-Cy7), CD7 (clone M-T701, Alexa700), CD123 (clone 6H6, Alexa700), CD11c (clone 3.9, Alexa700), CD20 (clone 2H7, PE-Cy5), IgA (goat polyclonal antibodies, APC), IgG (clone G18-145, BUV737), IgM (clone G20-127, BUV396), IgD (goat polyclonal antibodies, PE), CD80 (clone L307.4, BV786), CD95 (clone DX2, BV711), CD27 (clone M-T271, BUV563), CD21 (clone B-ly4, BV605), CD14 (clone M5E2, BV570) and CD138 (clone DL-101, PE-CF594).
Validation	mAbs were used according to manufacturer's instructions and previously published methods; mAbs were validated and titrated for specificity prior to use

Eukaryotic cell lines

Policy information about cell lines

Cell line source(s)	HEK293T and Vero E6 cells Commerically purchased (ATCC)
Authentication	Cell lines were not authenticated.
Mycoplasma contamination	Negative for mycoplasma

Commonly misidentified lines (See ICLAC register)	No commonly misidentified cell lines were used in the study

Animals and other organisms

Policy information about studies involving animals; ARRIVE guidelines recommended for reporting animal research

Laboratory animals	18 male and female cynomolgus macaques (Macaca fascicularis), 3-20 years old
Wild animals	None
Field-collected samples	None
Ethics oversight	All animal studies were conducted in compliance with all relevant local, state, and federal regulations and were approved by the Bioqual Institutional Animal Care and Use Committee (IACUC).

Note that full information on the approval of the study protocol must also be provided in the manuscript.
